# Sensory processing deficits and related cortical pathological changes in Alzheimer’s disease

**DOI:** 10.3389/fnagi.2023.1213379

**Published:** 2023-08-15

**Authors:** Nicole K. Zhang, Selena K. Zhang, Li I. Zhang, Huizhong W. Tao, Guang-Wei Zhang

**Affiliations:** ^1^Zilkha Neurogenetic Institute, Keck School of Medicine, University of Southern California, Los Angeles, CA, United States; ^2^Biomedical Engineering Program, Viterbi School of Engineering, University of Southern California, Los Angeles, CA, United States; ^3^Department of Physiology & Neuroscience, Keck School of Medicine, University of Southern California, Los Angeles, CA, United States

**Keywords:** Alzheimer’s disease, sensory processing, sensory cortex, cognitive function, pathology, amyloid-beta deposition, tauopathy, neuronal loss

## Abstract

Alzheimer’s disease (AD) is a progressive neurodegenerative disorder primarily affecting cognitive functions. However, sensory deficits in AD start to draw attention due to their high prevalence and early onsets which suggest that they could potentially serve as diagnostic biomarkers and even contribute to the disease progression. This literature review examines the sensory deficits and cortical pathological changes observed in visual, auditory, olfactory, and somatosensory systems in AD patients, as well as in various AD animal models. Sensory deficits may emerge at the early stages of AD, or even precede the cognitive decline, which is accompanied by cortical pathological changes including amyloid-beta deposition, tauopathy, gliosis, and alterations in neuronal excitability, synaptic inputs, and functional plasticity. Notably, these changes are more pronounced in sensory association areas and superficial cortical layers, which may explain the relative preservation of basic sensory functions but early display of deficits of higher sensory functions. We propose that sensory impairment and the progression of AD may establish a cyclical relationship that mutually perpetuates each condition. This review highlights the significance of sensory deficits with or without cortical pathological changes in AD and emphasizes the need for further research to develop reliable early detection and intervention through sensory systems.

## Introduction

Alzheimer’s disease (AD) is a progressive neurological disorder characterized by the degeneration of cognitive functions or dementia, with an often overlooked but significant impact on sensory modalities such as vision, hearing, smell and touch (Albers et al., 2015; Claire, 2019; Wiesman et al., 2021a). The sensory deficits commonly observed in Alzheimer’s patients, including decreased visual contrast sensitivity (Risacher et al., 2013), hearing loss (Uhlmann et al., 1986), and olfaction deficits (Claire, 2019), can substantially impair an individual’s quality of life and hinder their ability to carry out daily tasks. Many studies have indicated earlier onsets of sensory deficits as compared to the cognitive decline in AD (Peters et al., 1988; Cronin-Golomb et al., 1995; Devanand et al., 2000), including young-onset atypical AD (Graff-Radford et al., 2021), suggesting that these sensory impairments may be suitable to serve as early diagnostic biomarkers. Meanwhile, interventions based on sensory stimulation have demonstrated a potential in alleviating clinical symptoms in patients with AD (Smith and D’Amico, 2020). However, the exact mechanisms that contribute to sensory impairments in AD progression remain elusive.

In AD brains, pathological changes have been observed in sensory cortices (Ahmad et al., 2016; Brewer and Barton, 2016; Claire, 2019; Aylward et al., 2020), making it crucial to investigate the causal relationship between cortical pathology and central sensory impairments in AD patients. The cortical pathological alterations encompass the accumulation of extracellular amyloid-beta (Aβ) plaques (Braak et al., 1989) and intracellular neurofibrillary tangles (Lewis et al., 1987), as well as neuronal loss, synaptic dysfunction, and inflammation. The cerebral cortex is responsible for various higher-order functions, including the processing and integration of sensory information (Gilbert, 1983; Zatorre et al., 2002), and thus becomes an essential area of focus when studying the central sensory deficits in AD. For example, a strong correlation between the visual contrast sensitivity deficit and cerebral amyloid and tau depositions has been proposed (Risacher et al., 2020). Examining the cortical pathology underlying central sensory impairments in AD has not only led to a better understanding of the disease’s progression and its impact on sensory functions, but also paved the way for the development of early diagnosis tools and targeted therapeutic interventions to improve the overall quality of life of Alzheimer’s patients.

This literature review is focused on the central sensory processing deficits observed in Alzheimer’s patients and animal models and their associated cortical pathological changes, and thereby aims to offer a more holistic understanding of the disease’s impact on sensory modalities.

## Central sensory processing deficits in Alzheimer’s disease

### Visual perception deficits

**FIGURE 1:**
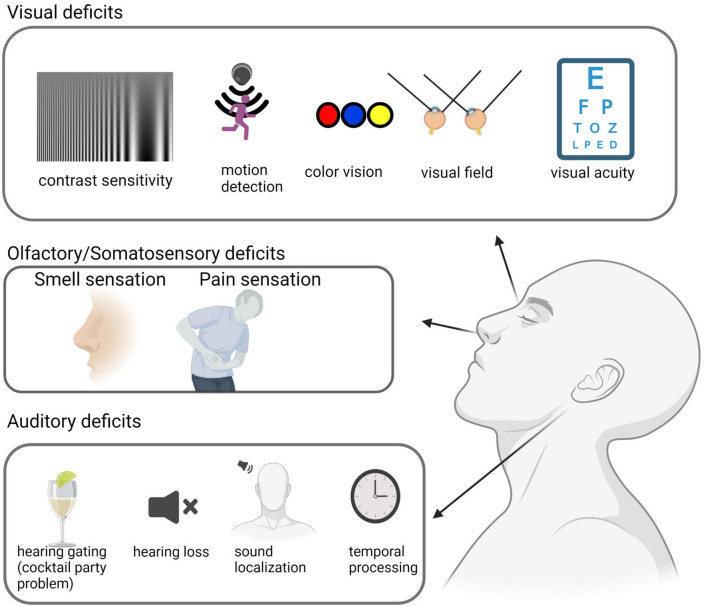
Sensory deficits in AD patients. Sensory deficits across sensory modalities are observed in AD patients. Visual deficits include decreased contrast sensitivity and motion detection, color blindness, visual field deficits, and a reduction in visual acuity. Auditory deficits include a decrease in hearing gating, hearing loss, and deficiencies in temporal processing and sound localization. Olfactory and somatosensory deficits consist of olfactory sensitivity and discrimination dysfunction, as well as a higher pain threshold. This illustration was generated using bioRender by N.Z. with approved license.

Various visual tests have been conducted in AD patients to assess contrast sensitivity, motion detection, visual field topography, visual acuity, and color vision (Figure 1). Although there are some discrepancies regarding some specific symptoms across different studies, it is generally agreed that some visual functions are impaired in AD patients compared to age-matched healthy controls (Bublak et al., 2011). Meanwhile, higher-order visual functions, such as contrast sensitivity and conscious moving object detection, appear to be more affected compared to basic visual functions such as visual acuity. See Table 1 for the prevalence.

**TABLE 1:** Prevalence of sensory deficit in AD.

Sensory deficit	Prevalence in AD	Disease stage	Age	References
Contrast sensitivity	47%	Dementia (MMSE 15–18)	40–90 years	Gilmore and Whitehouse, 1995
Color blindness	50%	Mild-severe dementia	84 years	Sadun et al., 1987
Visual evoked potentials abnormality	100%	Dementia (MMSE 23)	73–75 years	Fernandez et al., 2007
Visual Field Loss	61%	Dementia	73 years	Trick et al., 1995
Visual Acuity deficit	37%	Dementia (MMSE 18.7)	81 years	Marquié et al., 2019
Hearing gating	N/A	Dementia (MMSE 17.5)	71.2 years	Jessen et al., 2001
Hearing loss	31.7%	Early AD stages	55–75.5 years	Livingston et al., 2017
Odor identification deficit	85–90%	Dementia (MMSE 22)	75 years	Woodward et al., 2017

#### Contrast sensitivity

Contrast sensitivity (CS) tests measure the ability of an individual to distinguish visual patterns of varying spatial frequencies vs. background at different contrast levels (Cronin-Golomb et al., 1987). Many studies have consistently found deficiencies of CS in AD patients (Cronin-Golomb et al., 1991; Cronin-Golomb, 1995), with CS reduced at both low and high spatial frequencies (Cronin-Golomb et al., 1991). In addition, the CS deficits start from pre-clinical AD stages, indicating that they are sensitive to early AD-associated changes and could be a promising biomarker for diagnosis and monitoring (Risacher et al., 2013).

#### Motion detection

Visual motion perception is the ability to perceive and process movements in the visual field. It is an important aspect of visual processing that allows individuals to detect and respond to moving objects in their environment. Motion perception deficits have been reported in AD. Compared to healthy elderly adults, AD patients exhibit abnormal visual evoked potential to visual motion stimulation (Fernandez et al., 2007), and significantly higher thresholds to perceive motion signals (Gilmore et al., 1994; Kurylo et al., 1994), and the deficit is worsened with dementia severity (Gilmore et al., 1994). However, the motion perception deficit may reflect a cognitive decline. While the threshold of conscious perception of motion is significantly elevated the unconscious motion detection threshold remains normal (Silverman et al., 1994). Meanwhile, in a complex motion image perception test which requires participants to perceive shapes defined by motion cues, individuals with AD perform significantly worse than healthy controls, whereas the detection of motion direction *per se* is spared (Rizzo and Nawrot, 1998).

#### Color vision

Color blindness, or the inability to distinguish between certain colors, can arise in AD, as demonstrated by studies using various tests (Cronin-Golomb et al., 1991; Festa et al., 2005; Polo et al., 2017). The Ishihara test requires participants to recognize the pattern defined by color cues, and the PV-16 test requires participants to identify the most closely matched color. AD patients make more mistakes on both tests compared to healthy controls (Pache et al., 2003). The cognitive decline in AD also significantly impacts the ability of color discrimination. A study using the Farnsworth–Munsell 100 hue test to assay color discrimination has shown that AD patients make more errors than healthy controls, even after adjustments for cognitive performance (Salamone et al., 2009). The most errors are made in the blue-green chromatic areas and in naming colors, although the preference rank order of colors remains unaffected by disease severity (Wijk et al., 1999).

#### Visual field

The visual field refers to the area in which objects can be seen by an individual’s eyes without moving their head or eyes. AD patients exhibit visual field deficits (Armstrong, 1996). One study using the Humphrey automated perimetry to measure differential luminance sensitivity has found that AD patients exhibit a significant reduction in visual sensitivity globally throughout the visual field, with the deficit most pronounced in the inferonasal and inferotemporal arcuate regions of the visual field, and that the visual field loss tends to progress over time (Trick et al., 1995).

#### Visual acuity

Visual acuity is the ability of the eyes to detect and resolve fine details. In AD, visual acuity can be affected due to changes in the retina and visual cortex. Although a few studies have reported that AD patients have lower visual acuity than healthy controls (Sadun et al., 1987; Polo et al., 2017), most studies have not found significant differences of visual acuity between AD patients and healthy controls using the Snellen chart (Schlotterer et al., 1984; Katz and Rimmer, 1989; Mendez et al., 1990; Cronin-Golomb et al., 1991).

### Auditory perception deficits

Impaired central auditory processing has been found at early and mid-phases of AD (Strouse et al., 1995), often preceding the onset of clinical dementia (Gates et al., 2002). Meanwhile, hearing loss has been proposed as a significant risk factor for the development of AD (Griffiths et al., 2020), and the use of hearing aid shows some preventive effect (Livingston et al., 2020). Even for subclinical hearing loss, a study of 6,451 participants observed that cognitive function decreases with every 10 dB reduction in hearing (Golub et al., 2020). See Table 1 for the prevalence.

#### Hearing gating

Hearing gating refers to the brain’s ability to filter out irrelevant or redundant sound information in order to focus on important information and to protect against information overload. There is some evidence to suggest that hearing gating may be impaired in individuals with AD. Many studies using a double-click paradigm to measure sensory gating have shown that AD patients exhibit less inhibition to the response to the second click, which suggests a reduced ability of suppressing repeated auditory information (Jessen et al., 2001; Cancelli et al., 2006).

#### Hearing loss

Around 37% of AD patients show hearing loss (Livingston et al., 2017). The relationship between hearing loss and AD appears complex and intertwined. AD patients may have significantly worse hearing than normal elderly individuals (Gates et al., 2008). On the other hand, hearing loss *per se* has been proposed to facilitate the development of dementia (Griffiths et al., 2020). A prospective study involving 639 participants found that hearing loss and its severity are associated with an increased risk of developing dementia (Lin et al., 2011). Several possible underlying mechanisms have been proposed, including reduced sensory stimulation, or shared pathological factors (Griffiths et al., 2020). Regardless of the exact nature of the relationship, hearing loss can have a significant impact on the quality of life for individuals with AD, making it more difficult for them to communicate with caregivers and beloved ones, which contributes to feelings of isolation and depression. The use of hearing aid in the prevention of dementia has been investigated, showing some promising effects (Jiang et al., 2023).

A distinction between audibility and intelligibility should be noted. Audibility refers to the ability to hear sounds, typically assessed via an audiogram, which measures the quietest sounds an individual can hear at different frequencies. On the other hand, intelligibility refers to the ability to understand what is being heard, such as understanding speech in a noisy environment–also known as the “cocktail party problem.” Speech perception in noisy environments is impaired in some AD patients (Stevenson et al., 2022) that do not have hearing problems. Thus, AD brains may be less efficient at distinguishing auditory information from irrelevant noise.

#### Sound localization

Sound localization is a crucial aspect of auditory perception enabling individuals to recognize the source of sounds within their environment. This ability of spatial processing plays a significant role in navigation and communication. Sound localization is a core component of auditory scene analysis, which is vulnerable to AD (Goll et al., 2012). Studies have reported that AD patients exhibit a deficit in discrimination of stationary sound positions (Kurylo et al., 1993; Golden et al., 2015). The deficit, however, does not correlate with age or dementia severity (Kurylo et al., 1993), suggesting that it is not merely the result of a general cognitive decline, but rather indicative of specific impairments in the auditory processing system.

#### Temporal processing

Auditory temporal processing refers to the ability of the auditory system to perceive and process sound events that occur over time. This includes detection of changes in frequency, amplitude, and timing of sounds. The gap detection test has been used to assess temporal processing. At pre-clinical AD stages, patients start to show temporal processing through their difficulty in detecting silent gaps in continuous sounds (Iliadou et al., 2017). Meanwhile, individuals with AD may find it harder to discern rapidly occurring sound events or changes in the temporal structure of sounds (Stevenson et al., 2022). This also impacts their ability to understand speech, particularly in noisy environments.

### Other sensory perception deficits in Alzheimer’s disease

#### Olfactory

Alzheimer’s disease can affect various aspects of olfactory sensation, including olfactory sensitivity, discrimination and memory. It has been reported that the prevalence of olfactory dysfunction in AD could be up to 100% (Duff et al., 2002; Zou et al., 2016). Individuals with AD may experience a decline in their ability to detect and identify different odors (Albers et al., 2015). This reduction in olfactory sensitivity can affect the ability to enjoy food and to detect potentially dangerous situations (e.g., gas leaks or spoiled food). However, the olfactory deficit may be harder to notice compared to visual and auditory dysfunction, as it often goes undetected or underreported by patients and their caregivers. Nevertheless, the prevalence and early onset of olfactory dysfunction suggest a great potential of its usage as an early diagnostic marker of AD (Zou et al., 2016; Claire, 2019).

#### Somatosensory

Somatosensory deficits have received lower attention. While AD pathology spares the primary sensorimotor cortices (Uylings and de Brabander, 2002), somatosensory deficits have been reported in AD patients (Wiesman et al., 2021a). In particular, it has been well documented that AD patients exhibit a significantly higher pain threshold and diminished pain reaction compared to healthy controls (Kunz and Lautenbacher, 2004). However, the underlying mechanism remains elusive.

## Cortical pathology underlying sensory deficits in Alzheimer’s disease

**FIGURE 2:**
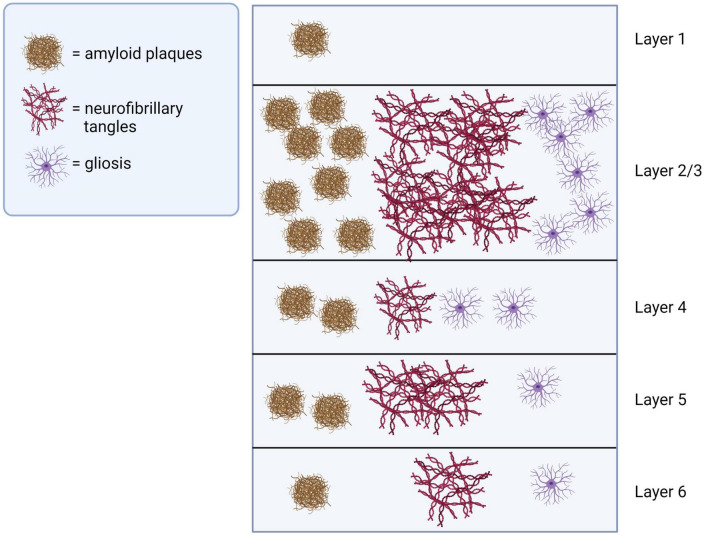
Pathological changes in sensory cortices of AD brains. Pathological changes including amyloid-beta plagues, neurofibrillary tangles and gliosis are observed across layers of sensory cortices in AD, with superficial layers 2 and 3 most severely affected. This illustration was generated using bioRender by N.Z. with approved license.

Sensory cortices are responsible for receiving and processing sensory information for generating perception. For example, the visual cortex, located in the occipital lobe at the back of the brain, receives visual information from the eyes and processes it into meaningful images. Similarly, the auditory cortex, located in the temporal lobe’s superior temporal gyrus, receives and processes auditory information from the ears. Neocortical pathological changes, including amyloid-beta (Aβ) deposition, tau pathology and neuronal loss, in areas of both primary and association cortices may underly the sensory perception deficits in AD brains (Figure 2). Understanding these changes is important for developing effective treatments for the sensory deficits associated with AD.

### Pathological changes in visual cortex

Alzheimer’s disease pathology, including amyloid plaques, neurofibrillary tangles and neurodegeneration, has been observed in the human occipital cortex (Braak et al., 1989; Armstrong et al., 1990; McKee et al., 2006; Crutch et al., 2012). Regarding the laminar distribution, some studies have demonstrated that layer (L)2/3 has the highest density of Aβ plaques (Duyckaerts et al., 1986; Braak et al., 1989; Armstrong, 2012), while others have reported high-density deposition L4 (Rafalowska et al., 1988), and L5 (Lewis et al., 1987). In early onset AD cases, the senile plaques are more frequently observed in the upper layers of the occipital cortex, and a change of visually evoked responses is suggested to be used as a diagnostic marker for early onset AD (Armstrong et al., 1990). Meanwhile, it has been consistently reported that the visual association area has a higher number of Aβ plaques compared to the primary visual area (Armstrong, 2012).

The neurofibrillary tangle (NFT) formation has also been observed in the visual cortical areas of AD brains (Lewis et al., 1987; McKee et al., 2006), although with a lower prevalence as compared to Aβ (27 vs. 72% of AD brains) (Armstrong et al., 1990). The NFT formation shows the highest density in L2/3 (Armstrong, 2012) and L5 (Lewis et al., 1987), and is significantly more abundant in visual association areas than the primary visual area (Lewis et al., 1987; Braak et al., 1989; Armstrong et al., 1990; Giannakopoulos et al., 2000; Armstrong, 2012). The difference is reported to be as high as 20-fold (Lewis et al., 1987). Another study (Leuba et al., 2001) has reported that the NFT formation is almost absent in the primary visual area in mild cognitive impairment (MCI), which is considered a precursor to AD.

Besides Aβ deposition and NFT formation, other pathological changes have also been reported in the visual cortex of AD brains. Extensive spine loss has been observed, together with pathology of apical and horizontal dendrites of neurons (Mavroudis et al., 2011). Cell loss has been reported with divergent vulnerability (Geneviève and Rudolf, 1994), although to a much lower degree compared to regions such as prefrontal and temporal association areas (Frisoni et al., 2007). Loss of SMI32-immunoreactive pyramidal neurons has been found in L4 of the primary visual cortex and L3 of the secondary visual cortex, while inhibitory interneurons, including parvalbumin, calretinin, and calbindin immunoreactive neurons, do not show any significant decrease in primary or secondary visual cortex (Leuba et al., 1998). Degeneration of cholinergic fibers in the visual cortex is not conclusive, as no deficit (Geula and Mesulam, 1996) and large depletion (Beach et al., 1992) have both been reported. Furthermore, gliosis occurs in the visual cortex, being the heaviest in primary visual cortex layers 2, 3, and 4 (Beach et al., 1988), and is potentially correlated with the plaque deposition (Geneviève and Rudolf, 1994). Neuropil threads without NFT formation have also been observed in L5 of the visual cortex (Braak et al., 1989). Moreover, inhibitory synapse loss is observed in the occipital lobe of AD patients, potentially contributing to the disruption of excitatory/inhibitory balance (Kurucu et al., 2022).

Visual association area has been suggested as one of the earliest brain regions showing pathological changes (senile plagues and NFT) in AD. The early display of pathology in the occipital cortex or its cortical thinning may be a potential biomarker for AD (Hwang et al., 2021). Studies using fMRI have suggested that higher-order visual association areas exhibit functional deficits earlier than the lower-order primary visual area (Huang et al., 2021). The early manifestation of AD pathology in the association cortex highlights its vulnerability (McKee et al., 2006). In comparison, the primary visual cortex is relatively spared (Brun and Gustafson, 1976; Cronin-Golomb et al., 1991; Iseri et al., 2006; Jack et al., 2008; Grothe et al., 2017). Nevertheless, an increase in the power of gamma oscillations, which are often associated with cognitive processes (Murty et al., 2018), has been observed in the primary visual cortex of AD patients during a visuospatial processing task (Wiesman et al., 2021b), suggesting functional deficits.

While there have been plenty of studies showing the presence of AD pathology in the visual cortex, how it contributes to the visual impairments remains largely unclear. Occipital atrophy has been associated with the visual hallucination (Holroyd et al., 2000) and the visual field deficit in AD (Armstrong, 1996). It has also been proposed that Aβ deposition leads to hyperactivity of the visual cortex, disrupting orientation tuning, functional connectivity and visual memory (Grienberger et al., 2012; L’Esperance et al., 2023; Niraula et al., 2023). A diminished cross-modal effect by activation of visual cortex observed in AD patients during a visual cue dependent task further suggests altered interactions between visual cortex and other cortical areas (Drzezga et al., 2005). Understanding how visual functional connectivity is altered may allow for specific impairments such as visuospatial dysfunction to serve as a potential biomarker for mild cognitive impairment (Yamasaki et al., 2012) and facilitate the development of non-invasive therapeutics (Martorell et al., 2019).

### Pathological changes in auditory cortex

Pathological changes in auditory pathways, including the auditory cortex, have been reported in AD patients (Sinha et al., 1993). In general, the changes in the auditory cortex share some similarities with the visual cortex. First, Aβ plaques are prevalent, as they have been reported in up to 89% of AD brains (Armstrong et al., 1990). Layers 2 and 3 show the highest density of Aβ deposition (Duyckaerts et al., 1986; Rafalowska et al., 1988). The associative area exhibits more severe pathological changes than the primary area (Esiri et al., 1986). The Aβ oligomer, closely correlated with the plaque deposition, has also been observed in the auditory cortex (Savioz et al., 2016). However, the pathological difference between primary and associative cortices may not correlate with clinical symptoms, as shown by a study comparing auditory performance associated with the primary auditory cortex (sound localization and perception of complex tones) and that associated with auditory association cortex (phoneme discrimination and tonal memory) (Kurylo et al., 1993).

Tau pathology is observed in the auditory cortex, being more severe in the associative area than the primary area (Esiri et al., 1986; Lewis et al., 1987; Arnold et al., 1991). Interestingly, chondroitin sulfate proteoglycans show high-level expression in the primary auditory cortex, but at lower levels in association areas, leading to a hypothesis that higher levels of these extracellular matrix proteins may be associated with less tau pathology (Brückner et al., 1999).

Neuronal loss is also a prominent pathological marker in the temporal cortex. Significantly decreased gray matter density has been found in the auditory association cortex but not the primary auditory cortex of AD patients (Aylward et al., 2020). Late-onset AD results in much greater atrophy of the temporal lobe, especially in the superior temporal gyrus, than early onset AD (Frisoni et al., 2007). A study using Golgi staining in the auditory cortex of AD brains has found that layer 1 Cajal–Rezis cells exhibit significant cell loss, together with dendritic loss and distortion (Baloyannis et al., 2007). Regarding the interneuron, it has been suggested that somatostatin-positive neurons decrease in the temporal cortex in AD, but not parvalbumin-positive neurons (Waller et al., 2020). The choline acetyltransferase activity, which is indicative of cholinergic neuronal transmission, shows a significant decrease in the auditory cortex (Esiri et al., 1990).

Gliosis has been observed in the auditory cortex of AD brains (Styren et al., 1998). In the lateral temporal cortex, the astrocytic glial fibrillary acidic protein (GFAP) increases with AD severity and correlates with amyloid and tau pathology (Buchanan et al., 2020), suggesting that gliosis could be a robust indicator of disease progression.

The dysfunction of several neurotransmitter systems has been demonstrated in the AD temporal cortex. Reduction of serotonin (5-HT) 2A receptors has been found to correlate with the decline in cognitive performance (Lai et al., 2005). Deficits of nicotinic receptors have been reported, although no agreement has been reached regarding the specific subunits that are impaired (Guan et al., 2001; Martin-Ruiz et al., 2002). GABA-related transcriptions, including the receptors and GABA-synthesizing enzymes, are downregulated in the temporal cortex (Govindpani et al., 2020), and the GABAA receptor α5 subunit is found to decrease based on immunostaining (Kwakowsky et al., 2018). The glutamate transporter-1 (GLT-1) in astrocytes, which is responsible for removing excessive glutamate, is significantly reduced (Hoshi et al., 2018), suggesting that glutamate neurotoxicity may play an important role in the neurodegeneration of AD. Moreover, postsynaptic density protein 95 (PSD-95), but not presynaptic synaptophysin, decreases progressively in the temporal cortex across the Braak stages of AD (Buchanan et al., 2020).

It should be noted that many of the studies have not specifically targeted the auditory cortex, but rather the larger temporal cortex (Armstrong et al., 1990; Waller et al., 2020). It is possible that some of the observations of neuronal loss and neurotransmission disruptions might be in fact in regions beyond the auditory cortex. Moreover, the relationship of AD pathology in auditory cortex to hearing dysfunction is still under debate.

### Pathological changes in the olfactory and somatosensory cortex

Amyloid beta and tau pathology has been observed in the olfactory bulb of individuals with AD as well as MCI (Kovács et al., 1999; Tsuboi et al., 2003; Attems and Jellinger, 2006; Albers et al., 2015). AD patients also show prominent atrophy in the primary olfactory cortex (Al-Otaibi et al., 2020). Meanwhile, fMRI studies have reported decreased activation of the primary olfactory cortex in AD patients passively presented with odors, while some other studies have found increased activation in early AD (Meadowcroft et al., 2019). It has been proposed that AD-related pathology begins within olfaction-related structures and subsequently spreads to additional brain regions and ultimately encompasses multiple areas of the brain (Braak and Braak, 1991; Claire, 2019).

Amyloid and tau pathology has also been observed in the somatosensory cortex in AD, and the pathological changes are more pronounced in the association area than the primary region (Arnold et al., 1991). On the other hand, cholinergic fibers remain relatively preserved in the somatosensory cortex of AD brains (Geula and Mesulam, 1996). Moreover, abnormal magnetoencephalography responses to somatosensory stimulation have been found in the primary somatosensory cortex in MCI (Stephen et al., 2010).

Overall, the cortical pathological changes share some similarities across different sensory modalities. The changes are in general more severe in sensory association areas than the primary sensory cortices, with superficial cortical layers exhibiting the most pronounced changes. This spatial bias of the pathological progression might possibly explain why the basic sensory functions (e.g., visual acuity, auditory frequency discrimination) are largely spared, while higher sensory functions (e.g., visual contrast sensitivity, hearing in a relatively noisy environment) exhibit deficits with early onsets.

## Sensory deficits and sensory cortical pathological changes in animal models of AD

Transgenic animals used in AD research may not recapitulate all sensory impairment phenotypes, however, they still provide valuable opportunities to understand the neuronal substrates underlying the disease. In the following section, we will summarize current understandings of sensory deficits and cortical pathological changes in AD-related animal models. See Table 2 for a comparison between human and animal studies.

**TABLE 2:** Comparison between human and animal studies.

Sense	Species	Sensory deficit	References
Visual	Human	Decline visual processing speed and short-term memory	Bublak et al., 2011
Visual	Human	Contrast sensitivity is reduced at both low and high spatial frequencies Color blindness—tests like the Ishihara test demonstrate an inability to distinguish specific colors	Cronin-Golomb et al., 1991
Visual	Human	AD patients have significantly higher thresholds to perceive motion; motion detection deficit	Gilmore et al., 1994
Visual	Human	Visual field sensitivity decreases, especially at inferonasal and inferotemporal arcuate regions	Trick et al., 1995
Visual	Human	No significant difference found in visual acuity between AD patients and healthy controls	Mendez et al., 1990
Auditory	Human	Less inhibition in response to the second click in the double-click paradigm; reduced hearing gating	Jessen et al., 2001
Auditory	Human	AD patients have significantly worse hearing than their regular elderly counterparts	Gates et al., 2008
Auditory	Human	Deficits in discrimination of stationary sound positions; impaired sound localization	Kurylo et al., 1993
Auditory	Human	Preclinical AD patients have difficulty with gap detection tests; signs of temporal processing deficit	Iliadou et al., 2017
Visual	5xFAD	Optomotor tests reveal impaired visual behavior at 6 months	Zhang et al., 2021
Visual	APP23xPS45	Impaired orientation tuning in the visual cortex	Grienberger et al., 2012
Visual	hAPP J20	Hyperactivity in visual cortex with disrupted functional connectivity; increased visually evoked response	Niraula et al., 2023
Auditory	APP/PS1	Hearing loss has been observed at whole-frequency range for 3–4 months	Liu et al., 2020
Auditory	5xFAD	ABR amplitude reduction	Na et al., 2023
Auditory	5xFAD	Acoustic startle response decrease and ABR threshold increases at 3–4 months	O’Leary et al., 2017
Auditory	APP/PS1	Reduction of pre-pulse inhibition at 7 and 22 months	Wang et al., 2012
Auditory	P301S	Enhancement of pre-pulse inhibition	Takeuchi et al., 2011
Auditory	5xFAD	Gap detection deficits progressively worsen from 2 months, especially for males	Weible et al., 2020

### Visual deficits in AD mouse models

Visual behavior has been examined in the 5xFAD mouse, which overexpresses human amyloid precursor protein (APP) and presenilin 1 (PS1), harboring five familial AD mutations and rapidly develops amyloid pathology. A study using an optomotor test, which measures the head movement following the direction of drifting gratings at various spatial frequencies, has found impaired visual behavior suggesting impaired contrast sensitivity at 6 months of age (Zhang et al., 2021). In addition, visual evoked potential (VEP) recording in the visual cortex has revealed abnormalities in visual acuity in young 5xFAD mice (Criscuolo et al., 2018). In rTg4510 mice, which exhibit tauopathy, the visually evoked pupil dilation was completely disrupted (Papanikolaou et al., 2022). Other visual related behavioral changes would require a further comprehensive characterization.

### Auditory deficits in AD mouse models

Hearing loss has been reported in the APP/PS1 mouse, which expresses a chimeric mouse/human amyloid precursor protein and a mutant human presenilin 1. As assessed by auditory brainstem response (ABR), hearing loss first appears at high frequency ranges as early as 2 months old and then extends to low frequency ranges. By 3–4 months of age, hearing loss is observed within the whole-frequency range (Liu et al., 2020).

In the 5xFAD mouse, it has been suggested that central gain increases preceding the reduction of ABR amplitudes (Na et al., 2023). The acoustic startle response (ASR) starts to decline at the age of 3–4 months, and ABR threshold increases at 13–14 months of age (O’Leary et al., 2017). The latency of ASR is prolonged as well (Story et al., 2019). In the TgCRND8 (overexpressing mutant human APP) mouse, the ASR consistently increases starting from 10-weeks old (McCool et al., 2003).

In the prepulse inhibition (PPI) test, APP/PS1 and TgCRND8 mice show a reduction in PPI at 7- and 22-month-old, respectively (McCool et al., 2003; Wang et al., 2012). However, in the P301S (mutant tau protein) mouse model, PPI is enhanced (Takeuchi et al., 2011).

The gap detection test has revealed temporal processing impairments in 5xFAD mice. The gap detection deficit is evident as early as approximately 2 months old and worsens over time. Interestingly, the impairment exhibits sex dimorphism, being more prominent and earlier (2-month) in males than females (Kaylegian et al., 2019; Weible et al., 2020). Meanwhile, both the neuronal responses to silent gaps in continuous white noise and the spontaneous firing in the auditory cortex are progressively reduced (Weible et al., 2020).

Together, these identified hearing deficits are suggested to serve as a potential early non-invasive detection biomarker in AD mouse models, since they exhibit an onset much earlier than cognitive impairments such as spatial learning deficits (Liu et al., 2020). Further research is necessary to explore the underlying neural mechanisms and determine whether the findings can be translated to human AD cases.

### Pathological changes in visual cortex of AD mice

Amyloid plaques have been observed in the visual cortex of 5xFAD and APP/PS1 mice, including the primary and secondary visual cortex (Whitesell et al., 2019; Zhang et al., 2021; Tsui et al., 2022). In the 5xFAD mouse, Aβ pathology starts from layer 5 of the cortex, due to the usage of Thy1 as the promotor, at 2 months old (Oakley et al., 2006). At 9 months old, Aβ deposition can be observed in layers 4, 5, and 6 (Tsui et al., 2022). Gliosis surrounding the Aβ plaques has been found as well (Soula et al., 2023). In the rTg4510 transgenic mouse, which is a tauopathy model, the neurofibrillary tangles emerge in the visual cortex between 2 and 4 months of age. Meanwhile, short- and long-term visual plasticity, as assessed by local field potential responses to a repeated visual stimulus, are disrupted at both early (5-month) and late (8-month) stages of tauopathy (Papanikolaou et al., 2020). These results indicate that tau pathology can also affect intrinsic cortical plasticity. A relatively comprehensive investigation of the post-weaning developmental stages in the 5xFAD mouse has shown that APP overloading occurs in L5 pyramidal neurons of the primary visual cortex during the critical period (4–5 weeks) for visual cortical plasticity (Chen et al., 2022).

Functional changes in the visual cortex have also been investigated. Using *in vivo* two photon calcium imaging, an increase in spontaneous activity and a reduction in visual and motor triggered signals in V1 have been observed in APP/PS1 mouse (Liebscher et al., 2016). In hAPP mouse, hyperactivity in visual cortex has also been observed, likely attributed to the increased excitatory-inhibitory synapse ratio (Niraula et al., 2023). Meanwhile, in the hAPP mouse, the visually evoked activity increased, which is different from the observation in APP/PS1 mouse (Niraula et al., 2023). In rTg4510 transgenic mouse, which develops tauopathy, the visual plasticity were found disrupted at early stages in V1 (Papanikolaou et al., 2022).

### Pathological changes in auditory cortex of AD mice

Similar to visual cortex, in the 5xFAD mouse, amyloid plaques are found in auditory cortex, including the primary and ventral (secondary) auditory cortex (Tsui et al., 2022; Weible and Wehr, 2022). At 9 months old, Aβ deposition is observed in layers 4, 5, and 6 (Tsui et al., 2022). It is worth noting that Aβ deposition profiles are variable between different animal models. For example, in APP/PS1 mice, Aβ plaques are observed more in the dorsal auditory cortex (Whitesell et al., 2019).

In the 3xTg-AD mouse, which is a triple transgenic model containing three mutations associated with familial Alzheimer’s disease, it has been found that hearing loss induced by noise exposure before the disease phenotype is manifested causes persistent synaptic and morphological alterations in the auditory cortex (Paciello et al., 2021). This is associated with earlier increased tau phosphorylation, neuroinflammation, and redox imbalance. The results suggest that hearing loss could potentially accelerate the neurodegeneration onset.

### Other sensory deficits and cortical pathology in AD animal models

In the olfactory system of the APP/PS1 model, Aβ deposition has been observed in the olfactory epithelium, olfactory bulb and olfactory cortex (Wu et al., 2013). In the Tg2576 mouse that overexpresses a mutant form of APP with the Swedish mutation, non-fibrillar Aβ deposition has been found within the olfactory bulb at 3 months of age, earlier than the deposition within any other brain region, and found to be in correlation with olfactory deficits (Wesson et al., 2010). This suggests that non-fibrillar rather than fibrillar Aβ-related mechanisms might contribute to early olfactory perceptual loss in AD. However, it shall be noted that, the Tg2576 mouse model exhibits non-fibrillar Aβ deposition not only in the olfactory bulb (Wesson et al., 2010) but also in the hippocampus (Alcantara-Gonzalez et al., 2021) as early as 2–3 months of age.

In the 5xFAD mouse, amyloid plaques can be observed in the somatosensory cortex (Ali et al., 2019), including primary and secondary somatosensory cortex. At 9 months old, Aβ deposition is observed in layers 4, 5, and 6 of the somatosensory cortex (Tsui et al., 2022). Plaques are more and larger with increasing cortical depths (Ali et al., 2019). There is significant spine loss on basal dendrites of neurons in somatosensory cortices of 6-month-old females (Crowe and Ellis-Davies, 2014). Regarding the interneuron, parvalbumin neurons show a significant loss at 6–9 months old, which is more prominent in the deeper layers (Ali et al., 2019). In the barrel cortex of APP/PS1 mice, Aβ pathology exhibits some spatial specificity, with plaques more concentrated in septal areas than barrels (Beker et al., 2012). Using voltage-sensitive dye imaging, a study has found abnormal sensory responses evoked by whisker deflections in the barrel cortex: both the amplitude and spatial spread of the responses are larger in transgenic than in control mice (Maatuf et al., 2016).

### The potential problems of using animal models in studying sensory deficits in AD

#### Heterogeneous phenotypes

Different animal models exhibit varying phenotypes. For example, hair cell degeneration has been observed in the 5xFAD (O’Leary et al., 2017) but not 3xTg-AD mouse (Wang and Wu, 2021). Amyloid plaques are observed in the inferior colliculus and medial geniculate body in the 5xFAD but not APP/PS1 mouse (Na et al., 2023). A reduction in PPI is observed in APP/PS1 and TgCRND8 mice (McCool et al., 2003; Wang et al., 2012), but PPI is enhanced in the P301S tau model (Takeuchi et al., 2011).

#### Potential confounding factors due to intrinsic sensory deficits

Certain mouse strains exhibit early onset sensory deficits, such as the C57 line, which displays age-related hearing loss. This can complicate the interpretation of different experimental results. Therefore, it is important to design appropriate control experiments to account for these factors.

#### Discrepancy in pathological changes between mouse models and human AD patients

In humans, the deposition of amyloid-beta is reported to be significantly higher in associative regions compared to the primary sensory cortex. However, this distinction is not replicated in mouse models. The distribution of amyloid beta is partially affected by the promotor being adopted for generating transgenic mice, e.g., the Thy1 promotor (Oakley et al., 2006) primarily drives expression of transgenes in layer 5 of the cortex without a bias toward primary or secondary cortical regions. Meanwhile, previous studies have suggested that neuronal activity could modulate the spatial distribution of the plaque deposition (Bero et al., 2011), with higher activity correlated with denser A-beta deposition. This is consistent with the idea of the “default-mode network” and predicts that brain regions with highest levels of spontaneous activity show the most prominent amyloid plaque (Greicius et al., 2004). However, we shall acknowledge that many more factors may contribute to the regional specificity of amyloid deposition in AD patients, which requires further investigation.

## Peripheral contribution to sensory deficits in AD

Although this review focuses on the central pathological changes, we shall point out that peripheral changes could also contribute to the sensory dysfunction observed in AD.

A recent study has revealed AD biomarkers, such as A-beta and microgliosis, in the retinal tissue from individuals experiencing MCI and early stage AD, which correlate with cognitive scores (Koronyo et al., 2023). Alterations in pattern electroretinograms (PERG) and visual evoked potentials (VEP) have been found in AD patients, suggesting the involvement of alterations of retinal ganglion cells in the progression of AD (Sartucci et al., 2010). In 5xFAD mice, inner retina impairment assessed by Aβ accumulation and PERG has been observed as early as 1 month of age, earlier than the emergence of VEP and visual acuity impairments (Criscuolo et al., 2018).

In the peripheral auditory system, inner and outer hair cells are significantly reduced in number at 15–16 months of age in 5xFAD (O’Leary et al., 2017) but not in 3xTg-AD mouse (Wang and Wu, 2021). Meanwhile, spiral ganglion neurons undergo significant degeneration in the 3xTg-AD mouse (Wang and Wu, 2021).

## Mechanisms linking cortical pathological changes and sensory deficits in AD

### Hyperactivity

Hyperactivity in both neurons and astrocytes brain has been documented in AD brains (Busche et al., 2008; Kuchibhotla et al., 2009; Wright et al., 2013; Busche and Konnerth, 2015; Targa Dias Anastacio et al., 2022), and the hyperactive neurons are found spatially close to amyloid beta deposition in the double transgenic APP23xPS45 mouse (Busche et al., 2008). Moreover, it has been proposed that hyperactivity could induce synaptic dysfunction and loss at early stages of AD (Busche et al., 2008). Regarding sensory processing, hyperactivity increases background neural activity and thus reduces signal-to-noise ratio of sensory responses, which may impair the neural circuit’s ability to accurately distinguish relevant sensory information from irrelevant background noise.

### Hypoactivity

Reduced neuronal activity and decreased metabolism have also been observed in AD pathology (Silverman et al., 2001; Busche et al., 2008). A synaptic failure theory is proposed to explain this functional deficit (Selkoe, 2002). Soluble tau has been suggested to be able to suppress neuronal activity in an *in vivo* two-photon Ca^2+^ imaging study (Busche et al., 2019). In sensory systems, reduced neuronal activity may directly result in functional impairment. For example, the reduced visually evoked activity in V1 in APP/PS1 mice (Liebscher et al., 2016) is associated with visuomotor integration deficits in AD.

### Synapse loss

The synaptic loss is highly correlated with functional deficits in AD (Terry et al., 1991) and is proposed as an early event (Masliah et al., 1990). Microglia may be a major player through synapse pruning (Rajendran and Paolicelli, 2018). Oligomeric A-beta species may impair the function and structure of synapses as well (Sheng et al., 2012). The synaptic loss can also lead to diminished functional connectivity between cortical neurons and cortical areas. This may underlie various sensory processing deficits observed in AD.

### Unbalanced excitatory-inhibitory (E-I) dynamics

Inhibitory neurons are particularly susceptible to AD (Petrache et al., 2019), leading to a decrease in the inhibitory tone. In APP23 mice, application of diazepam reduces the activity of hyperactive neurons, suggesting that reduced inhibition underlies hyperactivity (Busche et al., 2008). The reduced inhibition results in hyperexcitability of excitatory neurons, which may not only interfere with normal sensory signal processing but also exacerbate neuronal degeneration. Inhibitory neurons themselves may exhibit different activity changes. In the APP/PS1 mouse, cortical L2/3 somatostatin neurons in proximity to amyloid beta show increased activity, while parvalbumin neurons show decreased activity (Algamal et al., 2022). In the hippocampus, it has been shown Aβ-induced hyperexcitability of parvalbumin neurons leads to memory impairment although no changes are observed yet in intrinsic properties of pyramidal neurons (Hijazi et al., 2020). Thus, alterations in excitatory-inhibitory balance may greatly affect sensory processing in the cortex (Wood et al., 2017).

### Soluble amyloid-beta and tau toxicity

While primary cortices appear to be relatively spared from amyloid plaques and NFT formation, soluble amyloid-beta and tau have been proposed to be able to induce hyperactivity (Kopeikina et al., 2012; Hector and Brouillette, 2021), adding another layer to the complex pathogenesis of AD.

### Neurodegeneration

The death of neurons within sensory cortices can result in the disintegration of circuits necessary for normal sensory information processing, contributing to sensory deficits.

### Reduced sensory stimulation

Peripheral deficits can result in a reduction of sensory information relayed to cortical neurons, further exacerbating the sensory deficit.

### Non-sensory cortical central contribution

It has also been proposed that pathological changes in other central regions may contribute to sensory deficits. For instance, deficits in the CA1 region of the hippocampus have been suggested to contribute to deficits in the acoustic startle response in the Tg4-42 mouse (Sichler et al., 2019).

### Impaired cross-model suppression

In AD patients, cross-model suppression is progressively impaired (Drzezga et al., 2005), which may contribute to the dysfunction of sensory processing.

## Sensory deficit could be a driving force of AD progression

We propose that sensory impairment and the progression of AD may establish a cyclical relationship that mutually perpetuates each condition. Sensory deficits can result in partial sensory deprivation, a state that has been demonstrated to escalate spontaneous cortical activity (Zaforas et al., 2021). This heightened neuronal activity could in turn augment the secretion of amyloid-beta (Bero et al., 2011). The disrupted neuronal function could further exacerbate sensory deficits, creating a detrimental feedback loop. A deeper understanding of this cycle could lead to new approaches to preventing or slowing the progression of AD. Interventions designed to compensate for or mitigate sensory deficits could potentially break the cycle, thereby reducing cortical hyperactivity and the consequent overproduction of amyloid-beta. For example, hearing loss is a modifiable risk factor, and the use of hearing aid has shown some potential in attenuating the cognitive decline (Deal et al., 2015; Sarant et al., 2020), although with controversies (Jiang et al., 2023). The sensory system is readily accessible via non-invasive stimulation methods, offering a promising avenue for therapeutic interventions. Various trials utilizing external sensory stimulation have been conducted, yielding a mixed range of both positive (Iaccarino et al., 2016) and negative outcomes (Soula et al., 2023). These diverse results underscore the need for continued exploration and nuanced understanding of the potential impacts and optimal conditions for such interventions.

## Discussion and conclusion

This review has highlighted the growing body of evidence for sensory deficits and cortical pathological changes in Alzheimer’s disease, both in human subjects and animal models. As our understanding of the disease advances, it is crucial to consider the implications of these findings for early detection, diagnosis, and potential therapeutic interventions.

Firstly, there is an urgent need for early diagnosis of the disease. The early onset of sensory deficits (Claire, 2019) suggests their potential usage as biomarkers for early AD detection. As hearing loss and other sensory impairments can precede cognitive impairment, researchers should further explore the usage of non-invasive auditory tests in combination with other biomarkers to improve the accuracy and timeliness of AD diagnosis. Additionally, the development of techniques to assess other sensory domains, such as visual event-related potentials (vERPs), may provide valuable insights into the pathological progression of the disease.

Secondly, the mapping of cortical pathological changes in AD-related animal models, including amyloid-beta deposition, tauopathy, and alterations in neuronal excitability and synaptic function, has important implications for understanding the neural circuit mechanisms underlying sensory deficits. Future research should focus on characterizing the spatial and temporal progression of the pathological changes and their relationships to specific sensory deficits. This will help to clarify whether the observed sensory deficits are direct consequences of AD pathology or secondary effects related to the disease process.

Thirdly, the potential relationship between sensory loss and AD progression should also be considered. Studies have suggested that sensory deprivation or impairment, such as noise-induced hearing loss, may accelerate neurodegeneration in AD. This highlights the importance of examining the potential benefits of sensory-based interventions, such as hearing aids or cochlear implants, in delaying or mitigating AD progression.

Lastly, this review has emphasized the need for translational research that bridges the gap between animal model and human studies. While animal models offer invaluable insights into the cellular and molecular mechanisms underlying AD pathology, their direct translation to human patients should be approached with caution. For example, the laminar-specific pathological changes and their differences between primary and secondary cortical regions are not necessarily recapitulated in animal models.

Several key questions in the field remain unanswered. Firstly, a comprehensive evaluation of sensory deficits across a range of commonly used AD mouse models is lacking. This lack of systematic data makes it challenging to draw a clear picture of how sensory deficits manifest across different model systems. Secondly, the causal relationship between sensory deficits and AD pathology remains largely undefined. Many studies to date have concentrated on identifying correlations between sensory deficits and AD pathology, but the underlying causal mechanisms are yet not well understood. Thirdly, the precise cellular mechanisms contributing to these deficits remain largely speculative. While various theories have been proposed, concrete evidence to support these hypotheses is often lacking. Finally, much of the existing research has focused on singular sensory modalities, overlooking the potential impacts on multimodal sensory integration. Given the severe pathology observed in associative regions in AD, it is plausible that the integration of sensory information across multiple modalities may be particularly affected. However, this critical aspect of sensory processing in AD remains underexplored. Future research should aim to address these gaps to provide a more comprehensive understanding of sensory deficits in AD.

In conclusion, the exploration of sensory deficits and cortical pathological changes in AD has the potential to significantly advance our understanding of the disease, improve early detection and diagnosis, and inform the development of novel therapeutic interventions. Continued research in this area is essential for ultimately improving the lives of those affected by this devastating disorder.

## Author contributions

NZ and GZ primarily contributed to the manuscript. SZ helped with literature collection. HT and LZ helped with discussion and editing of the manuscript. All authors contributed to the article and approved the submitted version.
